# Causas Múltiplas de Morte Associadas à Parada Cardiorrespiratória Pediátrica de 1996 a 2019 no Brasil

**DOI:** 10.36660/abc.20230480

**Published:** 2024-04-09

**Authors:** Thayanne Mendes de Andrade, Mariara Lopes da Costa Marques,, Thaís Rocha Salim, Glaucia Maria Moraes de Oliveira

**Affiliations:** 1 Universidade Federal do Rio de Janeiro Rio de Janeiro RJ Brasil Programa de Pós-graduação em Cardiologia, Universidade Federal do Rio de Janeiro, Rio de Janeiro, RJ – Brasil; 2 Universidade de Vassouras Rio de Janeiro RJ Brasil Universidade de Vassouras, Rio de Janeiro, RJ – Brasil

**Keywords:** Causas Múltiplas de Morte, Parada Cardiorrespiratória Pediátrica, Brasil

## Abstract

**Fundamento:**

Em pediatria, a parada cardiorrespiratória (PCR) está associada a alta mortalidade e graves sequelas neurológicas. Informações sobre as causas e mecanismos de morte abaixo de 20 anos poderiam fornecer subsídios teóricos para a melhoria da saúde de crianças e adolescentes.

**Objetivos:**

Realizar uma análise populacional das taxas de mortalidade por causas primárias e múltiplas de morte abaixo de 20 anos, em ambos os sexos, no período de 1996 a 2019, no Brasil, e identificar a frequência com que a PCR foi registrada nas declarações de óbito (DOs) desses indivíduos e os locais de ocorrência dos óbitos, a fim de promover estratégias para melhorar a prevenção de mortes.

**Método:**

Estudo ecológico de séries temporais de óbitos em indivíduos menores de 20 anos, no período de 1996 a 2019, avaliando as taxas de mortalidade (TMs) e a mortalidade proporcional (MP) por causa básica de morte. Foram analisados os percentuais de PCR registrados em qualquer linha da DO e o local de ocorrência dos óbitos. Foram calculadas as TMs por 100 mil habitantes e a MP por causa básica de morte nos menores de 20 anos segundo sexo e faixa etária, os percentuais de óbito por causas básicas por faixa etária quando a PCR foi descrita em qualquer linha das Partes I e II da DO, e o percentual de óbitos por causas básicas segundo o local de ocorrência. Os dados foram retirados do DATASUS, IBGE e SINASC.

**Resultados:**

De 1996 a 2019, ocorreram 2.151.716 óbitos de menores de 20 anos, no Brasil, gerando uma taxa de mortalidade de 134,38 por 100 mil habitantes. A taxa de óbito foi maior entre os recém-nascidos do sexo masculino. Do total de óbitos, 249.334 (11,6%) tiveram PCR registrada em qualquer linha da DO. Especificamente, a PCR foi registrada 49.178 vezes na DO na faixa etária entre 1 e 4 anos e em 88.116 vezes entre 29 e 365 dias, correspondendo, respectivamente, a 26% e 22% dos óbitos nessas faixas etárias. Essas duas faixas etárias apresentaram as maiores taxas de PCR registradas em qualquer linha da DO. As principais causas básicas de óbito quando a PCR foi registrada na sequência de óbitos foram doenças respiratórias, hematológicas e neoplásicas.

**Conclusão:**

As causas perinatais e externas foram as principais causas de morte, com maior TM nos menores de 20 anos no Brasil de 1996 a 2019. Quando consideradas as causas múltiplas de morte, as principais causas primárias associadas à PCR foram as doenças respiratórias, hematológicas e neoplásicas. A maioria dos óbitos ocorreu no ambiente hospitalar. Melhor compreensão da sequência de eventos nesses óbitos e melhorias nas estratégias de ensino em ressuscitação cardiopulmonar pediátrica são necessárias.

**Figure:**
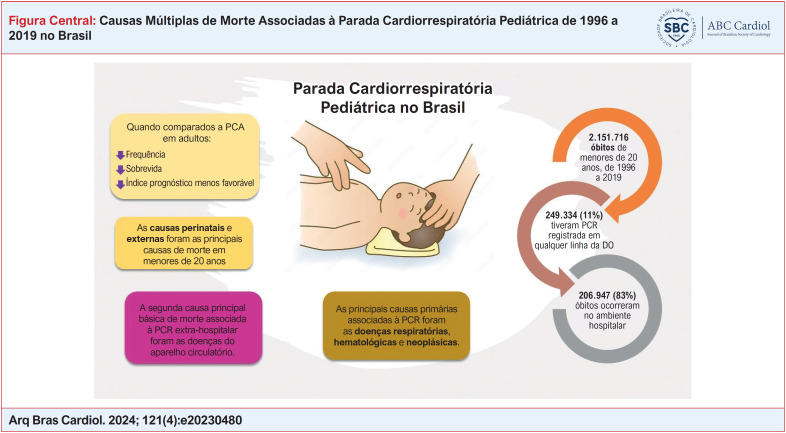
DO: declaração de óbito; PCA: parada cardiorrespiratória.

## Introdução

Nas últimas duas décadas, observou-se uma redução de quase 50% na ocorrência de óbitos nos menores de 20 anos em todo o mundo. No mesmo período e faixa etária no Brasil, a taxa de mortalidade caiu de 303,9 para 140,2 por 100 mil habitantes. Essa diminuição da mortalidade pode ser atribuída à redução das causas infecciosas de morte e à melhoria da saúde e da promoção da assistência.^1 - 4^ Ainda assim, a maioria desses óbitos poderia ter sido evitada por meio de ações que envolvessem diagnóstico, tratamento precoce e reversibilidade do mecanismo final de morte, a parada cardiorrespiratória (PCR).^1^

A etiologia da PCR difere entre as populações pediátrica e adulta, com maior mortalidade e sequelas neurológicas graves na primeira. Em pacientes pediátricos, os eventos de PCR ocorrem principalmente nos menores de 1 ano de idade e estão associados a uma taxa de mortalidade de 46,8%. Notavelmente, as chances de reverter um evento de PCR diminuem com a idade, com as taxas de mortalidade aumentando de 58,8% em crianças de 1 a 2 anos para 70% entre indivíduos de 12 a 17 anos.^2^ Ainda, na pediatria, a PCR ocorre mais frequentemente no ambiente hospitalar. Estudos internacionais relataram taxas de sobrevida na alta hospitalar após PCR de 32% a 40% em crianças, indicando que, na maioria dos casos, a morte não poderia ter sido evitada mesmo em um ambiente com recursos disponíveis para reversão e tratamento da PCR, apontando preparo inadequado dos profissionais responsáveis.^3 , 4^

Informações elucidando as causas e os mecanismos de morte em menores de 20 anos poderiam fornecer subsídios teóricos para a melhoria da saúde de crianças e adolescentes e aumentar as taxas de reversibilidade da PCR, uma vez conhecidas as condições mais associadas a esse evento. Estudos sobre o tema estão disponíveis na literatura, mas incluíram amostras pequenas, e nenhum analisou a associação entre PCR e doenças existentes antes da PCR, fatores limitantes para intervir e reverter um evento de PCR.^5 , 6^

Dessa forma, o objetivo deste estudo foi realizar uma análise populacional para compreender as taxas de mortalidade por causas primárias e múltiplas de morte nos menores de 20 anos, em ambos os sexos, no período de 1996 a 2019 no Brasil, e identificar a frequência de PCR registrada nas declarações de óbito (DOs) desses indivíduos e os locais de ocorrência dos óbitos, a fim de promover estratégias para melhorar a prevenção de mortes.

## Material e Métodos

Estudo ecológico de séries temporais de óbitos ocorridos de 1996 a 2019 nos menores de 20 anos no Brasil, avaliando as taxas de mortalidade e a mortalidade proporcional por causa básica de morte. Foram analisados os percentuais de PCR registrados em qualquer linha da DO e o local de ocorrência dos óbitos.

Além de conter informações básicas de identificação do indivíduo, a DO é composta por duas partes. A Parte I, composta por quatro linhas (a, b, c e d), descreve a doença que causou diretamente o óbito e as causas antecedentes, por meio da causa primária, intermediária e imediata do óbito. A Parte II descreve outras condições que não entraram na sequência do óbito, mas que contribuíram para essa morte.

No presente estudo, os dados referentes aos óbitos foram obtidos do Sistema de Informações sobre Mortalidade (SIM), disponível no site do Departamento de Informática do Sistema Único de Saúde (DATASUS).^7^ Esses conjuntos de informações compõem uma combinação de todas as DOs registradas no Brasil de 1996 a 2019, ano a ano, por Unidade da Federação. As causas básicas de morte foram registradas utilizando-se códigos da Classificação Estatística Internacional de Doenças e Problemas Relacionados à Saúde, 10ª Revisão (CID-10) da Organização Mundial da Saúde.^8^ Todos os arquivos foram convertidos para análise por meio do software Tab for Windows, versão 4.15 (DATASUS). Os dados, de ambos os sexos, foram coletados nas seguintes faixas etárias: (1) neonatos (até 28 dias de vida), (2) 29 a 365 dias de vida, (3) 1 a 4 anos, (4) 5 a 9 anos, (5) 10 a 14 anos e (6) 15 a 19 anos, seguindo o padrão proposto pela Organização Mundial da Saúde.

As informações populacionais, que foram utilizadas no estudo para o cálculo das taxas de mortalidade, são projeções de cálculos estatísticos realizados pelo Instituto Brasileiro de Geografia e Estatística (IBGE).^9^ Eles são baseados em censos, que estão disponíveis de 1980 a 2050 por macrorregião brasileira, sexo, faixa etária e por totais. Foram utilizadas projeções de 1996 a 2019 referentes às faixas etárias de 0 a 4 anos (excluindo nascidos vivos ocorridos no período), 5 a 9 anos, 10 a 14 anos e 15 a 19 anos, em ambos os sexos e em cada estado brasileiro. Para as idades menores de 1 ano, utilizou-se o número de nascidos vivos disponível no Sistema de Informações sobre Nascidos Vivos (SINASC).^10^

O estudo foi realizado de acordo com os princípios éticos e, por se fundamentar em bases de dados nacionais não identificadas disponíveis no site do DATASUS, foi dispensado de aprovação pelo comitê de ética e pesquisa, de acordo com a resolução 466/2012.

Foram utilizados os seguintes códigos da CID-10 para causas preveníveis por meio de medidas adequadas de prevenção e controle da saúde e atenção às doenças infecciosas e não transmissíveis: doenças infecciosas e parasitárias (A15 a A18, G00.1 a G03, L02 a L08, J00 a J06, J10 a J18, J20 a J22, N70 a N76, N39.0, I00 a I09, A00 a A09, A20 a A28, A30, A50 a A59, A63 a A64, A90 a A99, A75 a A79, A82, B03, B15, B17 a B19, B20 a B24, B50 a B83, B90 e B99), neoplasias (todos os códigos do Capítulo II) e doenças do sangue (todos os códigos do Capítulo III), doenças endócrinas (E01 a E05, E10 a E14 e E66), perturbações mentais (F00 a F99), doenças do sistema nervoso (todos os códigos do Capítulo VI), doenças do aparelho circulatório (Capítulo IX, agrupados em febre reumática I00 a I09, doenças hipertensivas I10 a I15, cardiopatias isquêmicas I20 a I25, cardiopatias pulmonares I26 a I28, doenças do pericárdio I30 a I32, doenças valvares I33 a I39, miocardite I40 a I41, cardiomiopatias I42 a I43, distúrbios de condução I44 a I49, insuficiência cardíaca I50, complicações de doenças cardíacas I51 a I52, doenças cerebrovasculares I60 a I69, doenças vasculares I70 a I89, distúrbios não especificados do sistema circulatório I95 a I99) e parada cardíaca (I46, I46.0, I46.1 e I46.9).^11 , 12^ Quanto às causas primárias, foram utilizados todos os códigos dos capítulos I, II, III, IV, V, VI, VII, IX, X, XI, XII, XIII, XIV, XVI, XIX, XX, XXI e XXII e os códigos Q00 a Q18, Q30 a Q99 e Q20 a Q28.9.

Foram calculadas as taxas de mortalidade por 100 mil habitantes e a mortalidade proporcional por causa básica de morte, segundo sexo e faixa etária inferior a 20 anos. Também foram estimados, por faixa etária, os percentuais de óbitos por causas básicas quando a PCR foi registrada em qualquer linha das Partes I e II da DO e os percentuais de óbitos por causas básicas, segundo o local de ocorrência, quando a PCR foi registrada na sequência de óbitos. Os locais de óbito foram agrupados em ocorridos (1) no ambiente hospitalar, para óbitos ocorridos em hospital ou outro serviço de saúde; (2) fora do ambiente hospitalar, para óbitos ocorridos no domicílio, em via pública ou em local desconhecido; e (3) perdas, quando o local do óbito não foi mencionado na DO.

Vale ressaltar que a PCR é um mecanismo de morte e deve ter etiologia atribuída, que deve ser descrita como causa básica de morte na DO. Portanto, a PCR foi considerada neste estudo como um evento descrito na DO e assistida pelo médico declarante, a fim de avaliar sua ocorrência na sequência de óbitos. Analisamos o registro da PCR utilizando os códigos I46, I46.0, I46.1 e I46.9 em qualquer uma das linhas das Partes I (a, b, c e d) e II da DO. Ao final da análise, todas as causas registradas independentemente em cada linha foram somadas para análise das causas múltiplas de morte quando a PCR foi descrita em qualquer linha das Partes I e II.

As análises foram realizadas utilizando-se os programas Microsoft Excel^13^ e Stata, versão 14.^14^

## Resultados

De 1996 a 2019, ocorreram 2.151.716 óbitos de menores de 20 anos no Brasil, gerando uma taxa de mortalidade de 134,38 por 100 mil habitantes. A taxa de óbito foi maior entre os recém-nascidos do sexo masculino, independentemente da causa básica de morte. Do total de óbitos, 249.334 (11,6%) tiveram a PCR registrada em qualquer linha da DO, como mostra a Figura Central . Especificamente, a PCR foi registrada 49.178 vezes na DO na faixa etária entre 1 e 4 anos e em 88.116 vezes entre 29 e 365 dias, correspondendo, respectivamente, a 26% e 22% dos óbitos nessas faixas etárias. Essas duas faixas etárias apresentaram as maiores taxas de PCR registradas em qualquer linha da DO.

As Tabelas 1 e 2 mostram as taxas de mortalidade e a mortalidade proporcional por causa básica de óbito segundo faixa etária no sexo masculino e feminino, respectivamente. Entre as causas básicas de morte no sexo masculino, os maiores percentuais de mortalidade proporcional foram as causas perinatais em neonatos e as doenças infecciosas e parasitárias nos menores de 1 ano, exceto para os neonatos. As causas externas de morte predominaram nas demais faixas etárias. No sexo feminino, as doenças do aparelho respiratório foram pronunciadas entre as idades de 1 a 4 anos, e as causas externas foram as principais causas de morte acima dos 5 anos.

**Tabela 1:** Mortalidade proporcional e taxas de mortalidade segundo grupos de causas primárias e de idade, em menores de 20 anos, no sexo masculino, no Brasil, de 1996 a 2019

Causas primárias de morte		< 20 anos total (ambos os sexos)	Sexo masculino						
			Total (sexo masculino)	Neonatos	< 1 ano (exceto os neonatos)	1-4	5-9	10-14	15-19
Doenças infecciosas e parasitárias	Óbitos	138.769	76.575	3.410	42.800	13.895	4.292	3.802	6.269
	MP (%)	6,45	5,71	0,77	19,50	13,52	7,51	4,96	1,83
	Mort 100K	8,67	9,44	9,29	116,66	9,17	2,11	1,87	2,98
Neoplasias e doenças do sangue	Óbitos	87.674	49.146	524	4.779	9.437	8.868	8.904	13.593
	MP (%)	4,07	3,67	0,12	2,18	9,18	15,52	11,61	3,97
	Mort 100K	5,47	6,06	1,43	13,03	6,23	4,37	4,39	6,46
Doenças endócrinas	Óbitos	39.102	20.420	675	11.216	4.007	1.228	1.132	1.674
	MP (%)	1,82	1,52	0,15	5,11	3,90	2,15	1,48	0,49
	Mort 100K	2,44	2,52	1,84	30,57	2,65	0,60	0,56	0,79
Doenças do sistema nervoso	Óbitos	63.439	36.536	874	8.675	8.038	4.856	5.215	7.631
	MP (%)	3,04	2,72	0,20	3,95	7,82	8,50	6,80	2,23
	Mort 100K	3,96	4,50	2,38	23,64	5,30	2,39	2,57	3,62
Doenças do aparelho respiratório	Óbitos	139.334	76.603	2.749	39.093	17.026	4.295	4.202	7.456
	MP (%)	6,48	5,71	0,62	17,81	16,57	7,51	5,48	2,18
	Mort 100K	8,70	9,44	7,49	106,55	11,24	2,12	2,07	3,54
Doenças perinatais	Óbitos	666.901	376.961	355.309	20.360	442	81	69	69
	MP (%)	30,99	28,12	80,24	9,27	0,43	0,14	0,09	0,02
	Mort 100K	41,65	46,46	968,43	55,49	0,29	0,04	0,03	0,03
Outras MC	Óbitos	*128.910*	66.687	11.040	4.526	4.313	1.234	963	933
	MP (%)	5,99	4,97	2,49	2,06	4,20	2,16	1,26	0,27
	Mort 100K	8,05	8,22	30,09	12,34	2,85	0,61	0,47	0,44
MAC	Óbitos	85.943	46.049	21.757	17.328	4.066	1.076	802	875
	MP (%)	3,99	3,43	4,91	7,89	3,96	1,88	1,05	0,25
	Mort100K	*5,37*	5,67	59,30	47,23	2,68	0,53	0,39	0,41
DAC	Óbitos	*43.522*	24.546	720	4.381	2.830	1.932	3.548	8.807
	MP (%)	*2,06*	1,83	0,16	2,00	2,75	3,38	4,63	2,57
	Mort 100K	*2,72*	3,02	1,96	11,94	1,86	0,95	1,75	4,18
Causas externas	Óbitos	558.684	458.427	2.631	12.451	22.847	22.569	39.917	275.896
	MP (%)	*25,96*	34,20	0,59	5,67	22,23	39,49	52,06	80,68
	Mort 100K	34,89	56,50	7,17	33,94	15,08	11,12	19,67	131,06
Mal definidas	Óbitos	*146.220*	84.476	11.085	35.246	12.192	4.385	5.047	12.965
	MP (%)	6,80	6,30	2,50	16,06	11,86	7,67	6,58	3,79
	Mort 100K	9,13	10,41	30,21	96,07	8,05	2,16	2,49	6,16
**Todas as causas**	Óbitos	2.151.716	1.340.345	442.800	219.531	102.753	57.146	76.669	341.961
	MP (%)	*100,0*	*100,0*	100,0	100,0	*100,0*	*100,0*	*100,0*	100,0
	Mort 100K	134,38	165,20	*1.206,89* (1)	*598,35* (1)	*67,85* (2)	28,15	37,77	162,44

DAC: doenças do aparelho circulatório; MAC: malformações do aparelho circulatório; Mort 100K: taxa de mortalidade por 100 mil; MP (%): mortalidade proporcional, percentual; Outras MC: outras malformações congênitas, excluindo as MAC.

(1) Mortalidade por 100 mil nascidos vivos

(2) Mortalidade por 100 mil indivíduos na população de 0 a 4 anos, excluindo os nascidos vivos.

**Tabela 2:** Mortalidade proporcional e taxas de mortalidade segundo grupos de causas primárias e de idade, em menores de 20 anos, no sexo feminino, no Brasil, de 1996 a 2019

Causas primárias de morte		< 20 anos total (ambos os sexos)	Sexo feminino						
			Total (sexo feminino)	Neonatos	< 1 ano (exceto os neonatos)	1-4	5-9	10-14	15-19
Doenças infecciosas e parasitárias	Óbitos	138.769	61.948	2.828	33.716	12.566	3.737	2.994	4.687
	MP (%)	6,45	7,71	0,83	18,84	14,55	9,09	6,26	5,27
	Mort 100K	8,67	7,84	8,10	96,57	8,28	1,91	1,48	2,28
Neoplasias e doenças do sangue	Óbitos	87.674	38.491	483	4.014	7.965	7.067	7.293	9.522
	MP (%)	4,07	4,79	0,14	2,24	9,23	17,19	15,25	10,72
	Mort 100K	5,47	4,87	1,38	11,50	5,25	3,62	3,60	4,64
Doenças endócrinas	Óbitos	39.102	18.625	491	9.482	3.877	1.055	1.180	2.026
	MP (%)	1,82	2,32	0,14	5,30	4,49	2,56	2,47	2,28
	Mort 100K	2,44	2,36	1,41	27,16	2,56	0,54	0,58	0,99
Doenças do sistema nervoso	Óbitos	63.439	26.878	586	6.652	6.627	3.874	4.147	4.340
	MP (%)	3,04	3,34	0,17	3,72	7,68	9,42	8,67	4,88
	Mort 100K	3,96	3,40	1,68	19,05	4,37	1,98	2,04	2,11
Doenças do aparelho respiratório	Óbitos	139.334	62.580	1.988	30.569	15.829	3.873	3.612	5.417
	MP (%)	6,48	7,79	0,59	17,08	18,33	9,42	7,55	6,10
	Mort 100K	8,70	7,92	5,69	87,55	10,43	1,98	1,78	2,64
Doenças perinatais	Óbitos	666.901	285.670	268.340	16.247	361	79	54	45
	MP (%)	30,99	35,55	79,16	9,08	0,42	0,19	0,11	0,05
	Mort 100K	41,65	36,17	768,56	46,53	0,24	0,04	0,03	0,02
Outras MC	Óbitos	*128.910*	60.318	12.530	4.653	4.098	1.157	985	771
	MP (%)	5,99	7,51	3,70	2,60	4,75	2,81	2,06	0,87
	Mort 100K	8,05	7,64	35,89	13,33	2,70	0,59	0,48	0,37
MAC	Óbitos	85.943	39.637	16.573	16.304	4.149	1.049	781	657
	MP (%)	3,99	4,93	4,89	9,11	4,80	2,55	1,63	0,74
	Mort 100K	*5,37*	5,02	47,47	46,70	2,73	0,54	0,38	0,32
DAC	Óbitos	*43.522*	19.618	504	4.064	2.760	1.773	3.033	5.881
	MP (%)	*2,06*	2,44	0,15	2,27	3,20	4,31	6,34	6,62
	Mort 100K	*2,72*	2,48	1,44	11,64	1,82	0,91	1,49	2,87
Causas externas	Óbitos	558.684	100.127	1.849	9.509	14.607	11.864	16.433	37.510
	MP (%)	*25,96*	12,46	0,55	5,31	16,92	28,85	34,36	42,22
	Mort 100K	34,89	12,68	5,30	27,23	9,63	6,07	8,10	18,28
Mal definidas	Óbitos	*146.220*	61.237	7.802	27.756	10.562	3.450	3.739	6.514
	MP (%)	6,80	7,62	2,30	15,51	12,23	8,39	7,82	7,33
	Mort 100K	9,13	7,75	22,35	79,50	6,96	1,77	1,84	3,17
**Todas as causas**	Óbitos	2.151.716	803.513	338.997	178.972	86.330	41.118	47.827	88.847
	MP (%)	*100,0*	*100,0*	*100,0*	100,0	*100,0*	*100,0*	*100,0*	*100,0*
	Mort 100K	134,38	101,73	*970,93 (1)*	*512,60 (1)*	56,91 (2)	21,05	23,58	43,32

DAC: doenças do aparelho circulatório; MAC: malformações do aparelho circulatório; Mort 100K: taxa de mortalidade por 100 mil; MP (%): mortalidade proporcional, percentual; Outras MC: outras malformações congênitas, excluindo as MAC.

(1) Mortalidade por 100 mil nascidos vivos

(2) Mortalidade por 100 mil indivíduos na população de 0 a 4 anos, excluindo os nascidos vivos.

**Figura 1:**
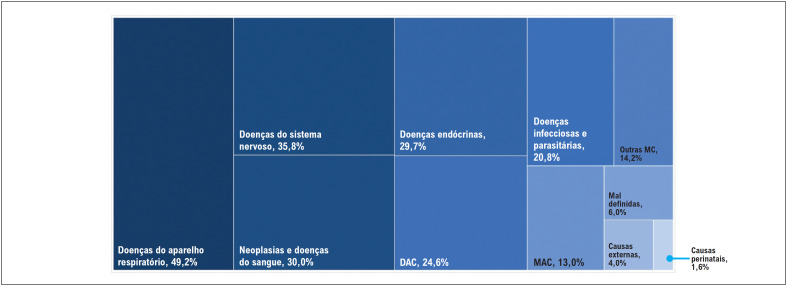
Taxas de causas múltiplas de óbito associadas à parada cardiorrespiratória em menores de 20 anos, no Brasil, de 1996 a 2019. DAC: doenças do aparelho circulatório; MAC: malformações do aparelho circulatório; Outras MC: outras malformações congênitas, excluindo as MAC.

**Figura 2:**
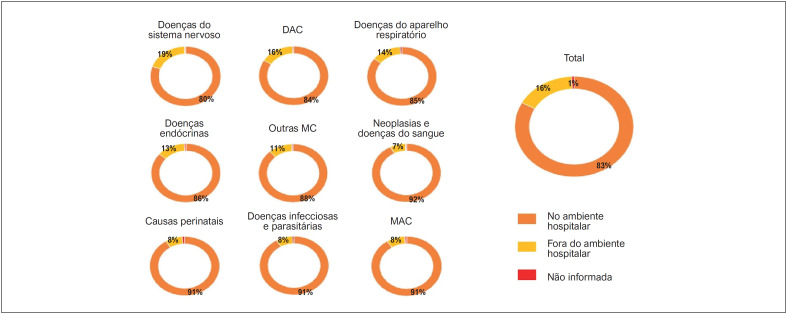
Taxas de causas múltiplas de óbito associadas à parada cardiorrespiratória segundo o local de ocorrência, no Brasil, de 1996 a 2019, em menores de 20 anos. DAC: doenças do aparelho circulatório; MAC: malformações do aparelho circulatório; Outras MC: outras malformações congênitas, excluindo as MAC.

A Figura 1 mostra as causas básicas de óbito quando a PCR foi registrada em qualquer linha da DO, ou seja, quando o indivíduo apresentou esse evento na sequência de óbito. As principais causas de morte no Brasil nos menores de 20 anos foram as doenças do aparelho respiratório, seguidas pelas doenças do sistema nervoso. No entanto, quando cada faixa etária foi analisada individualmente, observaram-se diferenças entre as causas básicas de óbito. Especificamente, as principais causas de morte foram as perinatais no período neonatal (Suplemento – Figura 1) e as doenças do aparelho respiratório em crianças menores de 5 anos (Suplemento – Figuras 2 e 3).

As principais causas básicas de morte quando a PCR foi registrada na sequência do óbito foram as doenças neoplásicas e hematológicas na faixa etária de 5 a 14 anos (Suplemento – Figuras 4 e 5) e as causas externas em adolescentes de 15 a 19 anos (Suplemento – Figura 6). Assim, em indivíduos maiores de 5 anos, a PCR ocorreu principalmente por causas não respiratórias.

Quando analisamos a frequência das causas básicas de óbito, segundo o local de ocorrência, quando a PCR foi registrada na sequência de óbitos, no mesmo período, no Brasil e nos menores de 20 anos de idade, observamos que 83% dos óbitos ocorreram no ambiente hospitalar, enquanto 16% ocorreram fora do ambiente hospitalar. Em 1% dos casos, o local do óbito não foi informado. As principais causas básicas de morte no ambiente hospitalar foram as neoplásicas e hematológicas, as malformações do aparelho circulatório e as doenças infecciosas e parasitárias, enquanto as que ocorreram fora do ambiente hospitalar foram as doenças do sistema nervoso e as doenças do aparelho circulatório, como mostra a Figura 2 .

## Discussão

Foram identificados quatro padrões de distribuição das principais causas básicas de morte nos menores de 20 anos de 1996 a 2019 no Brasil, quando a PCR foi registrada na sequência do óbito: causas perinatais no período neonatal, doenças do aparelho respiratório abaixo de 5 anos, doenças neoplásicas e hematológicas entre 5 e 14 anos, e causas externas em adolescentes de 15 a 19 anos. O principal local de ocorrência dos óbitos foi em ambiente hospitalar.

Apesar do declínio global da mortalidade infantil nas últimas décadas, particularmente com a redução do componente pós-neonatal, o componente neonatal sofreu poucas variações, o que se reflete na maior mortalidade proporcional por causas perinatais de óbito e maior taxa de mortalidade neonatal, em ambos os sexos, como encontramos no presente estudo.^15^ Maior mortalidade em jovens do sexo masculino também foi observada em outro estudo.^16^ No sexo masculino, as maiores taxas de mortalidade proporcional são as causas perinatais e externas, sendo que as causas externas ganham maior importância com o avançar da idade, o que pode ser explicado pela maior exposição desse sexo à violência interpessoal e aos acidentes automobilísticos.^1^

Quando analisados os óbitos em que a PCR foi registrada na sequência de óbito, as principais causas básicas de óbito foram as doenças respiratórias, neoplásicas e hematológicas, exceto para as faixas etárias incluindo neonatos e adolescentes de 15 a 19 anos, nas quais as principais causas foram perinatais e externas, respectivamente. Quando excluídas as causas perinatais e externas de óbito nessas faixas etárias, as causas respiratórias, neoplásicas e hematológicas voltaram a se destacar, sugerindo que a alta mortalidade por causas perinatais e externas, nas respectivas faixas etárias, pode ser fator de confusão, encobrindo as principais causas básicas de morte quando se registra PCR.

Assim, excluindo-se da análise as causas perinatais e causas externas de óbito, observaram-se dois padrões em que a PCR foi registrada na sequência de óbitos: causas respiratórias, principalmente abaixo de 5 anos, e causas neoplásicas e hematológicas, acima de 5 anos. As causas respiratórias também se destacaram como comorbidades pré-existentes em dois estudos prospectivos realizados no Malawi e nos EUA com populações comparáveis à do presente estudo em termos de faixa etária; no entanto, ambos os estudos incluíram uma pequena amostra restrita a um hospital terciário.^17 - 20^ Assim, é possível inferir que a presença de doenças respiratórias pode ser um fator de risco para PCR nessa faixa etária.

A maior taxa de causas neoplásicas e hematológicas acima de 5 anos pode ser explicada pelo aumento da incidência de PCR intra-hospitalar em pacientes pediátricos com doenças crônicas, com maior mortalidade entre as doenças oncológicas e hematológicas.^2 , 17^ Assim, pode-se inferir também que a presença de doenças neoplásicas e hematológicas nessa faixa etária pode ser um fator de risco para PCR.

Em relação aos óbitos extra-hospitalares associados à PCR, as principais causas básicas de morte foram as doenças do sistema nervoso e as doenças do aparelho circulatório. Esses dados corroboram os encontrados em estudo realizado na Austrália com indivíduos menores de 50 anos, que demonstrou que, em indivíduos menores de 18 anos, as principais causas básicas de morte associadas à PCR extra-hospitalar foram respiratória e doenças do aparelho circulatório.^21^ Isso mostra que precisamos explorar melhor os fatores de risco e as causas associadas à PCR nessa faixa etária.

Ainda em relação ao ambiente extra-hospitalar no Brasil, o desfecho está relacionado ao ritmo da PCR. Os ritmos chocáveis são responsáveis por 80% dos casos, com sobrevida de 50% a 70%. Enquanto os ritmos não chocáveis têm uma taxa de sobrevivência inferior a 17% em todas as idades. Em metanálise que incluiu 141 estudos realizados na América do Norte, Europa, Ásia e Oceania sobre PCR em adultos no ambiente extra-hospitalar, encontraram taxas de retorno à circulação espontânea de 29,7%, com sobrevida inferior a 10%.^22 - 27^ A literatura ainda carece de dados semelhantes relacionados à faixa etária pediátrica.

Chama a atenção a maior mortalidade em ambientes hospitalares, geralmente dotados de recursos estruturais e humanos para a realização de RCP. Estudo realizado nos EUA com a faixa etária pediátrica mostrou taxa de mortalidade superior a 60% por PCR em hospitais.^22^ Além disso, como mostrado em um estudo observacional brasileiro que incluiu cuidados de RCP realizados em nível individual e de equipe em um hospital pediátrico, há baixa adesão ao protocolo de Suporte Avançado de Vida em Pediatria entre os profissionais de saúde. Esse dado está alinhado com os resultados de mortalidade encontrados no presente estudo, uma vez que a qualidade da reanimação impacta diretamente na sobrevida desses indivíduos.^18^

A análise comparativa entre causas básicas de morte e PCR como evento notificado em amostra populacional é pioneira na literatura científica e permite o entendimento das comorbidades e etiologias mais associadas a esse evento, traçando um perfil de indivíduos com maior risco de apresentar PCR e evoluir para óbito, para que esses óbitos possam ser prevenidos. Ainda assim, é necessário conhecer a sequência mais comum de eventos que culmina em tais mortes para que possam ser prevenidas de forma mais específica.

As limitações deste estudo incluem o uso de dados secundários e DOs preenchidos com informações incompletas. No entanto, estudos recentes têm mostrado uma melhora na qualidade da codificação dos óbitos no Brasil e uma diminuição no uso do *garbage code* , principalmente nos últimos 20 anos.^23^ Esses são os dados disponíveis com maior impacto na saúde no Brasil, além de retratarem nossa população, justificando sua ampla utilização na literatura científica brasileira.

## Conclusão

As maiores taxas de mortalidade por causas básicas de morte nos menores de 20 anos no Brasil de 1996 a 2019 foram perinatal e causas externas. Quando avaliamos as causas múltiplas de morte, as principais causas primárias relacionadas à PCR foram as doenças respiratórias, hematológicas e neoplásicas. A maioria dos óbitos ocorreu no ambiente hospitalar. Faz-se necessária uma maior compreensão da cadeia de eventos desses óbitos e a ampliação e aprimoramento das estratégias de ensino em RCP pediátrica, voltadas principalmente para os profissionais de saúde.

## * Material suplementar

Para figura suplementar 1, por favor, clique aqui .

Para figura suplementar 2, por favor, clique aqui .

Para figura suplementar 3, por favor, clique aqui .

Para figura suplementar 4, por favor, clique aqui .

Para figura suplementar 5, por favor, clique aqui .

Para figura suplementar 6, por favor, clique aqui .
