# Supplementary material for: Causas Múltiplas de Morte Associadas à Parada Cardiorrespiratória Pediátrica de 1996 a 2019 no Brasil
Source: Arq Bras Cardiol. 2024 Apr 9;121(4):e20230480. [Article in Portuguese] doi: 10.36660/abc.20230480 (PMC11081088; doi:10.36660/abc.20230480)
Supplement: Translation

# Translation: Multiple Causes of Death Associated with Pediatric Cardiopulmonary Arrest from 1996 to 2019 in Brazil

**DOI:** 10.36660/abc.20230480i

**Authors:** Thayanne Mendes de Andrade, Mariara Lopes da Costa Marques, Thaís Rocha Salim, Glaucia Maria Moraes de Oliveira

**Affiliations:** 1 Rio de Janeiro RJ Brazil Programa de Pós-graduação em Cardiologia, Universidade Federal do Rio de Janeiro, Rio de Janeiro, RJ – Brazil; 2 Rio de Janeiro RJ Brazil Universidade de Vassouras, Rio de Janeiro, RJ – Brazil

**Keywords:** Multiple Causes of Death, Pediatric Cardiopulmonary Arrest, Brazil

## Abstract

**Background:**

In pediatrics, cardiopulmonary arrest (CPA) is associated with high mortality and severe neurologic sequelae. Information on the causes and mechanisms of death below the age of 20 years could provide theoretical support for health improvement among children and adolescents.

**Objectives:**

To conduct a population analysis of mortality rates due to primary and multiple causes of death below the age of 20 years in both sexes from 1996 to 2019 in Brazil, and identify the frequency in which CPA was recorded in the death certificates (DCs) of these individuals and the locations where the deaths occurred, in order to promote strategies to improve the prevention of deaths.

**Method:**

Ecological time-series study of deaths below the age of 20 years from 1996 to 2019, evaluating the mortality rates (MRs) and proportional mortality (PM) by primary cause of death. We analyzed the percentages of CPA recorded in any line of the DC and the location where the deaths occurred. We calculated the MRs per 100,000 inhabitants and the PM by primary cause of death under the age of 20 years according to sex and age group, the percentages of death from primary causes by age group when CPA was described in any line of Parts I and II of the DC, and the percentage of deaths from primary causes according to their location of occurrence. We retrieved the data from DATASUS, IBGE, and SINASC.

**Results:**

From 1996 to 2019, there were 2,151,716 deaths below the age of 20 years in Brazil, yielding a mortality rate of 134.38 per 100,000 inhabitants. The death rate was highest among male neonates. Of all deaths, 249,334 (11.6%) had CPA recorded in any line of the DC. Specifically, CPA was recorded in 49,178 DCs between the ages of 1 and 4 years and in 88,116 of those between the ages of 29 and 365 days, corresponding, respectively, to 26% and 22% of the deaths in these age groups. These two age groups had the highest rates of CPA recorded in any line of the DC. The main primary causes of death when CPA was recorded in the sequence of death were respiratory, hematologic, and neoplastic diseases.

**Conclusion:**

Perinatal and external causes were the primary causes of death, with highest MRs under the age of 20 years in Brazil from 1996 to 2019. When multiple causes of death were considered, the main primary causes associated with CPA were respiratory, hematologic, and neoplastic diseases. Most deaths occurred in the hospital environment. Better understanding of the sequence of events in these deaths and improvements in teaching strategies in pediatric cardiopulmonary resuscitation are needed.

**Figure:** CPA: cardiopulmonar arrest; DC: death certificate.

## Introduction

Over the last two decades, a decrease of almost 50% in the occurrence of deaths below the age of 20 years has been observed worldwide. In the same period and age group in Brazil, the mortality rate decreased from 303.9 to 140.2 per 100,000 inhabitants. This decrease in mortality can be attributed to a reduction in infectious causes of death and improvements in health and care promotion.^1 - 4^Still, most of these deaths could have been prevented through actions involving diagnosis, early treatment, and reversibility of the final mechanism of death, i.e., cardiopulmonary arrest (CPA).^1^

The CPA etiology differs between the pediatric and adult populations, with greater mortality and severe neurological sequelae in the former. In pediatric patients, CPA events occur mostly below the age of 1 year and are associated with a mortality rate of 46.8%. Notably, the odds of reversing from a CPA event decrease with age, with mortality rates increasing from 58.8% in children aged 1 to 2 years to 70% among individuals aged 12 to 17 years.^2^In pediatrics, CPA occurs more frequently in the hospital environment. International studies have reported survival rates on hospital discharge after CPA of 32% to 40% in children, indicating that, in most cases, death could not have been prevented even in an environment with available resources for CPA reversal and treatment, pointing out inadequate preparation of the professionals responsible for care.^3 , 4^

Information elucidating the causes and mechanisms of death under the age of 20 years could provide theoretical support for improving health in children and adolescents and increase the rates of CPA reversibility, once the conditions most associated with this event are known. Studies on this subject are available in the literature, but they have included small samples, and none have analyzed the association between CPA and existing diseases preceding the CPA, which are limiting factors for intervening and reversing a CPA event.^5 , 6^

Thus, the aim of this study was to carry out a population analysis to understand the mortality rates due to primary and multiple causes of death under the age of 20 years in both sexes from 1996 to 2019 in Brazil, and to identify the frequency of CPA recorded in the death certificates (DCs) of these individuals and the locations where the deaths occurred, in order to promote strategies to improve the prevention of deaths.

## Material and Methods

Ecological time-series study of deaths that occurred from 1996 to 2019 below the age of 20 years in Brazil, evaluating mortality rates and proportional mortality by primary cause of death. We analyzed the percentages of CPA recorded in any line of the DC, and the location where the deaths occurred.

In addition to containing basic information identifying the individual, the DC comprises two parts. Part I, consisting of four lines (a, b, c, and d), describes the disease that directly caused the death and the antecedent causes, through the primary, intermediate, and immediate cause of death. Part II describes other conditions that were not included in the sequence of the death but contributed to the death.

In the present study, the data related to the deaths were obtained from the Mortality Information System (SIM), available on the website of the Department of Informatics of the Unified Health System (DATASUS).^7^These sets of information comprise a combination of all the DCs recorded in Brazil from 1996 to 2019, year by year, by federative unit. The primary causes of death were recorded using codes from the International Statistical Classification of Diseases and Related Health Problems, 10^th^Revision (ICD-10) of the World Health Organization.^8^All files were converted for analysis using the software Tab for Windows, version 4.15 (DATASUS). The data, of both sexes, were collected from the following age groups: (1) neonates (up to 28 days of life), (2) 29 to 365 days of life, (3) 1 to 4 years, (4) 5 to 9 years, (5) 10 to 14 years, and (6) 15 to 19 years, following the standard proposed by the World Health Organization.

Population information, which was used in the study to calculate the mortality rates, is based on projections from statistical calculations performed by the Brazilian Institute of Geography and Statistics (IBGE).^9^They are based on censuses, which are available from 1980 to 2050 by Brazilian macro-region, sex, age group, and totals. We used projections from 1996 to 2019 related to the age groups 0 to 4 years (excluding live births that occurred in the period), 5 to 9 years, 10 to 14 years, and 15 to 19 years, in both sexes and in each Brazilian state. For ages below 1 year, we used the number of live births available in the information system on live births (SINASC).^10^

The study was carried out in accordance with ethical principles, and, since it was based on unidentified national databases available on the DATASUS website, it was exempt from approval by the ethics and research committee, in accordance with resolution 466/2012.

The following ICD-10 codes were used for causes that are preventable through appropriate measures promoting health prevention and control and attention to infectious and noncommunicable diseases: infectious and parasitic diseases (A15 to A18, G00.1 to G03, L02 to L08, J00 to J06, J10 to J18, J20 to J22, N70 to N76, N39.0, I00 to I09, A00 to A09, A20 to A28, A30, A50 to A59, A63 to A64, A90 to A99, A75 to A79, A82, B03, B15, B17 to B19, B20 to B24, B50 to B83, B90 and B99), neoplasms (all codes in Chapter II), and diseases of the blood (all codes in Chapter III), endocrine diseases (E01 to E05, E10 to E14, and E66), mental disorders (F00 to F99), diseases of the nervous system (all codes in Chapter VI), diseases of the circulatory system (Chapter IX, grouped into rheumatic fever I00 to I09, hypertensive diseases I10 to I15, ischemic heart diseases I20 to I25, pulmonary heart diseases I26 to I28, diseases of the pericardium I30 to I32, valve disorders I33 to I39, myocarditis I40 to I41, cardiomyopathies I42 to I43, conduction disorders I44 to I49, heart failure I50, complications of heart diseases I51 to I52, cerebrovascular diseases I60 to I69, vascular diseases I70 to I89, unspecified disorders of the circulatory system I95 to I99), and cardiac arrest (I46, I46.0, I46.1, and I46.9).^11 , 12^As for the primary causes, we used all codes from Chapters I, II, III, IV, V, VI, VII, IX, X, XI, XII, XIII, XIV, XVI, XIX, XX, XXI, and XXII and the codes Q00 to Q18, Q30 to Q99, and Q20 to Q28.9.

We calculated the mortality rates per 100,000 inhabitants and the proportional mortality by primary cause of death according to sex and age group below the age of 20 years. We also estimated by age group the percentages of death from primary causes when CPA was recorded in any line of Parts I and II of the DC and the percentages of death from primary causes, according to their location of occurrence, when CPA was recorded in the death sequence. The locations of death were grouped as occurring (1) in the hospital environment, for deaths that occurred in a hospital or another health care facility; (2) outside the hospital environment, for deaths that occurred at home, on a public street, or in an unknown location; and (3) losses, when the location of the death was not mentioned in the DC.

Of note, CPA is a mechanism of death and must have an attributed etiology, which should be described as the primary cause of death in the DC. Therefore, CPA was considered in this study as an event described in the DC and assisted by the declaring physician, in order to assess its occurrence in the death sequence. We analyzed the recording of CPA using the codes I46, I46.0, I46.1, and I46.9 in any of the lines of Parts I (a, b, c, and d) and II of the DC. At the end of the analysis, all causes recorded independently in each line were added up to analyze the multiple causes of death when CPA was described in any line of Parts I and II.

The analyses were performed using the software programs Microsoft Excel^13^and Stata, version 14.^14^

## Results

From 1996 to 2019, there were 2,151,716 deaths below the age of 20 years in Brazil, yielding a mortality rate of 134.38 per 100,000 inhabitants. The death rate was highest among male neonates, regardless of the primary cause of death. Of all deaths, 249,334 (11.6%) had CPA recorded in any line of the DC, as shown in the Central Figure . Specifically, CPA was recorded in 49,178 DCs between the ages of 1 and 4 years and in 88,116 of those between the ages of 29 and 365 days, corresponding, respectively, to 26% and 22% of the deaths in these age groups. These two age groups had the highest rates of CPA recorded in any line of the DC.

Tables 1 and 2 show the mortality rates and proportional mortality by primary cause of death according to age group in the male and female sex, respectively. Among the primary causes of death in the male sex, the highest percentages of proportional mortality were perinatal causes in neonates and infectious and parasitic diseases below the age of 1 year, excluding neonates. External causes of death predominated in the other age groups. In the female sex, diseases of the respiratory system were pronounced between the ages of 1 to 4 years, and external causes were the main causes of death above the age of 5 years.

**Table 1:** Proportional mortality and mortality rates according to groups of primary causes and age, below the age of 20 years, in the male sex, in Brazil from 1996 to 2019

Primary causes of deaths		< 20 years total (both sexes)	Male sex						
			Total (male sex)	Neonates	< 1 year (except for neonates)	1-4	5-9	10-14	15-19
Infectious and parasitic diseases	Deaths	138.769	76.575	3.410	42.800	13.895	4.292	3.802	6.269
	PM (%)	6.45	5.71	0.77	19.50	13.52	7.51	4.96	1.83
	Mort 100K	8.67	9.44	9.29	116.66	9.17	2.11	1.87	2.98
Neoplasms and diseases of the blood	Deaths	87.674	49.146	524	4.779	9.437	8.868	8.904	13.593
	PM (%)	4.07	3.67	0.12	2.18	9.18	15.52	11.61	3.97
	Mort 100K	5.47	6.06	1.43	13.03	6.23	4.37	4.39	6.46
Endocrine diseases	Deaths	39.102	20.420	675	11.216	4.007	1.228	1.132	1.674
	PM (%)	1.82	1.52	0.15	5.11	3.90	2.15	1.48	0.49
	Mort 100K	2.44	2.52	1.84	30.57	2.65	0.60	0.56	0.79
Diseases of the nervous system	Deaths	63.439	36.536	874	8.675	8.038	4.856	5.215	7.631
	PM (%)	3.04	2.72	0.20	3.95	7.82	8.50	6.80	2.23
	Mort 100K	3.96	4.50	2.38	23.64	5.30	2.39	2.57	3.62
Diseases of the respiratory system	Deaths	139.334	76.603	2.749	39.093	17.026	4.295	4.202	7.456
	PM (%)	6.48	5.71	0.62	17.81	16.57	7.51	5.48	2.18
	Mort 100K	8.70	9.44	7.49	106.55	11.24	2.12	2.07	3.54
Perinatal diseases	Deaths	666.901	376.961	355.309	20.360	442	81	69	69
	PM (%)	30.99	28.12	80.24	9.27	0.43	0.14	0.09	0.02
	Mort 100K	41.65	46.46	968.43	55.49	0.29	0.04	0.03	0.03
Other CM	Deaths	*128.910*	66.687	11.040	4.526	4.313	1.234	963	933
	PM (%)	5.99	4.97	2.49	2.06	4.20	2.16	1.26	0.27
	Mort 100K	8.05	8.22	30.09	12.34	2.85	0.61	0.47	0.44
MCS	Deaths	85.943	46.049	21.757	17.328	4.066	1.076	802	875
	PM (%)	3.99	3.43	4.91	7.89	3.96	1.88	1.05	0.25
	Mort 100K	*5.37*	5.67	59.30	47.23	2.68	0.53	0.39	0.41
DCS	Deaths	*43.522*	24.546	720	4.381	2.830	1.932	3.548	8.807
	PM (%)	*2.06*	1.83	0.16	2.00	2.75	3.38	4.63	2.57
	Mort 100K	*2.72*	3.02	1.96	11.94	1.86	0.95	1.75	4.18
External causes	Deaths	558.684	458.427	2.631	12.451	22.847	22.569	39.917	275.896
	PM (%)	*25.96*	34.20	0.59	5.67	22.23	39.49	52.06	80.68
	Mort 100K	34.89	56.50	7.17	33.94	15.08	11.12	19.67	131.06
Poorly defined	Deaths	*146.220*	84.476	11.085	35.246	12.192	4.385	5.047	12.965
	PM (%)	6.80	6.30	2.50	16.06	11.86	7.67	6.58	3.79
	Mort 100K	9.13	10.41	30.21	96.07	8.05	2.16	2.49	6.16
**All causes**	Deaths	2.151.716	1.340.345	442.800	219.531	102.753	57.146	76.669	341.961
	PM (%)	*100.0*	*100.0*	100.0	100.0	*100.0*	*100.0*	*100.0*	100.0
	Mort 100K	134.38	165.20	*1.206.89* (1)	*598.35* (1)	*67.85* (2)	28.15	37.77	162.44

DCS: diseases of the circulatory system; MCS: malformations of the circulatory system; Mort 100K: mortality rate per 100,000; Other CM: other congenital malformations excluding MCS; PM (%): proportional mortality, in percentage.

(1) Mortality per 100,000 live births,

(2) Mortality per 100,000 individuals in the population from 0 to 4, excluding live births.

**Table 2:** Proportional mortality and mortality rates according to groups of primary causes and age, below the age of 20 years, in the female sex, in Brazil from 1996 to 2019

Primary causes of deaths		< 20 years total (both sexes)	Female sex						
			Total (female sex)	Neonates	< 1 year (except for neonates)	1-4	5-9	10-14	15-19
Infectious and parasitic diseases	Deaths	138.769	61.948	2.828	33.716	12.566	3.737	2.994	4.687
	PM (%)	6.45	7.71	0.83	18.84	14.55	9.09	6.26	5.27
	Mort 100K	8.67	7.84	8.10	96.57	8.28	1.91	1.48	2.28
Neoplasms and diseases of the blood	Deaths	87.674	38.491	483	4.014	7.965	7.067	7.293	9.522
	PM (%)	4.07	4.79	0.14	2.24	9.23	17.19	15.25	10.72
	Mort 100K	5.47	4.87	1.38	11.50	5.25	3.62	3.60	4.64
Endocrine diseases	Deaths	39.102	18.625	491	9.482	3.877	1.055	1.180	2.026
	PM (%)	1.82	2.32	0.14	5.30	4.49	2.56	2.47	2.28
	Mort 100K	2.44	2.36	1.41	27.16	2.56	0.54	0.58	0.99
Diseases of the nervous system	Deaths	63.439	26.878	586	6.652	6.627	3.874	4.147	4.340
	PM (%)	3.04	3.34	0.17	3.72	7.68	9.42	8.67	4.88
	Mort 100K	3.96	3.40	1.68	19.05	4.37	1.98	2.04	2.11
Diseases of the respiratory system	Deaths	139.334	62.580	1.988	30.569	15.829	3.873	3.612	5.417
	PM (%)	6.48	7.79	0.59	17.08	18.33	9.42	7.55	6.10
	Mort 100K	8.70	7.92	5.69	87.55	10.43	1.98	1.78	2.64
Perinatal diseases	Deaths	666.901	285.670	268.340	16.247	361	79	54	45
	PM (%)	30.99	35.55	79.16	9.08	0.42	0.19	0.11	0.05
	Mort 100K	41.65	36.17	768.56	46.53	0.24	0.04	0.03	0.02
Other CM	Deaths	*128.910*	60.318	12.530	4.653	4.098	1.157	985	771
	PM (%)	5.99	7.51	3.70	2.60	4.75	2.81	2.06	0.87
	Mort 100K	8.05	7.64	35.89	13.33	2.70	0.59	0.48	0.37
MCS	Deaths	85.943	39.637	16.573	16.304	4.149	1.049	781	657
	PM (%)	3.99	4.93	4.89	9.11	4.80	2.55	1.63	0.74
	Mort 100K	*5.37*	5.02	47.47	46.70	2.73	0.54	0.38	0.32
DCS	Deaths	*43.522*	19.618	504	4.064	2.760	1.773	3.033	5.881
	PM (%)	*2.06*	2.44	0.15	2.27	3.20	4.31	6.34	6.62
	Mort 100K	*2.72*	2.48	1.44	11.64	1.82	0.91	1.49	2.87
External causes	Deaths	558.684	100.127	1.849	9.509	14.607	11.864	16.433	37.510
	PM (%)	*25.96*	12.46	0.55	5.31	16.92	28.85	34.36	42.22
	Mort 100K	34.89	12.68	5.30	27.23	9.63	6.07	8.10	18.28
Poorly defined	Deaths	*146.220*	61.237	7.802	27.756	10.562	3.450	3.739	6.514
	PM (%)	6.80	7.62	2.30	15.51	12.23	8.39	7.82	7.33
	Mort 100K	9.13	7.75	22.35	79.50	6.96	1.77	1.84	3.17
**All causes**	Deaths	2.151.716	803.513	338.997	178.972	86.330	41.118	47.827	88.847
	PM (%)	*100.0*	*100.0*	*100.0*	100.0	*100.0*	*100.0*	*100.0*	*100.0*
	Mort 100K	134.38	101.73	*970.93* (1)	*512.60* (1)	56.91 (2)	21.05	23.58	43.32

DCS: diseases of the circulatory system; MCS: malformations of the circulatory system; Mort 100K: mortality rate per 100 thousand; Other CM: other congenital malformations excluding MCS; PM (%): proportional mortality, in percentage.

(1) Mortality per 100,000 live births,

(2) Mortality per 100,000 individuals in the population from 0 to 4, excluding live births.

Figure 1 shows the primary causes of death when CPA was recorded in any line of the DC, i.e., when the individual presented this event in the death sequence. The main primary causes of death in Brazil below the age of 20 years were diseases of the respiratory system, followed by diseases of the nervous system. However, when each age group was analyzed individually, we observed differences between the primary causes of death. Specifically, the primary causes of death were perinatal causes in the neonatal period ( Supplement - Figure 1 ) and diseases of the respiratory system in children younger than 5 years ( Supplement - Figures 2 and 3 ).

**Figure 1:** Rates of multiple causes of death associated with cardiopulmonary arrest under the age of 20 years in Brazil form 1996 to 2019. DCS: diseases of the circulatory system; MCS: malformations of the circulatory system; OtherCM: other congenital malformations excluding MCS.

The primary causes of death when CPA was recorded in the sequence of the death were neoplastic and hematological diseases in the age group of 5 to 14 years ( Supplement - Figures 4 and 5 ) and external causes in adolescents aged 15 to 19 years ( Supplement - Figure 6 ). Thus, in individuals older than 5 years, CPA occurred mainly due to non-respiratory causes.

When we analyzed the frequency of primary causes of death according to the location of occurrence when CPA was recorded in the sequence of death, in the same period in Brazil and below the age of 20 years, we observed that 83% of the deaths occurred in the hospital environment, while 16% occurred outside the hospital environment. In 1% of the cases, the location of the death was not notified. The main primary causes of death in the hospital environment were neoplasms and diseases of the blood, malformations of the circulatory system, and infectious and parasitic diseases, while those occurring outside the hospital environment were diseases of the nervous system and diseases of the circulatory system, as shown in Figure 2 .

**Figure 2:** Rates of multiple causes of death associated with cardiopulmonary arrest according to the location of occurrence in Brazil, from 1996 to 2019, below the age of 20 years. DCS: diseases of the circulatory system; MCS: malformations of the circulatory system; OtherCM: other congenital malformations excluding MCS.

## Discussion

We identified the following four patterns of distribution of primary causes of death below the age of 20 years from 1996 to 2019 in Brazil when CPA was recorded in the sequence of the death: perinatal causes in the neonatal period, diseases of the respiratory system below the age of 5 years, neoplastic and hematologic diseases between the ages of 5 and 14 years, and external causes in adolescents aged 15 to 19 years. The main location of occurrence of the deaths was in a hospital environment.

Despite the global decrease in infant mortality in recent decades, particularly with a reduction in the post-neonatal component, the neonatal component has undergone few variations, which is reflected in the higher proportional mortality of perinatal causes of death and higher neonatal mortality rate, in both sexes, as we found in the present study.^15^Higher mortality in male youths has also been observed in another study.^16^In the male sex, the highest proportional mortality rates are perinatal and external causes, with external causes gaining greater importance with advancing age, which can be explained by the greater exposure of this sex to interpersonal violence and car accidents.^1^

When we analyzed the deaths in which CPA was recorded in the death sequence, the main primary causes of death were respiratory, neoplastic, and hematologic diseases, except for the age groups including neonates and adolescents aged 15 to 19 years, in whom the main causes were perinatal and external, respectively. When perinatal and external causes of death were excluded in these age groups, respiratory, neoplastic, and hematologic causes became prominent again, suggesting that a high mortality rate due to perinatal and external causes, in the respective age groups, may be confounding factors, covering up the main primary causes of death when CPA is recorded.

Thus, excluding the perinatal and external causes of death from the analysis, we observed two patterns in which CPA was recorded in the sequence of death: respiratory causes, especially below the age of 5 years, and neoplastic and hematological causes, above the age of 5 years. Respiratory causes also stood out as pre-existing comorbidities in two prospective studies conducted in Malawi and in the USA with populations comparable to that of the present study in terms of age groups; however, both these studies included a small sample restricted to a tertiary hospital.^17 - 20^Thus, it is possible to infer that the presence of respiratory diseases may be a risk factor for CPA in this age group.

The higher rate of neoplastic and hematologic causes above the age of 5 years may be explained by an increased incidence of in-hospital CPA in pediatric patients with chronic diseases, with the highest mortality among oncologic and hematological diseases.^2 , 17^Thus, we may also infer that the presence of neoplastic and hematologic diseases in this age group may be a risk factor for CPA.

Regarding out-of-hospital deaths associated with CPA, the main underlying causes of death were nervous system diseases and diseases of the circulatory system. These data corroborate those found in a study conducted in Australia with individuals under 50 years of age, which demonstrated that, in individuals under 18 years of age, the main underlying causes of death associated with out-of-hospital CPA were respiratory diseases and diseases of the circulatory system.^21^This shows that we need to further explore the risk factors and causes associated with CPA in this age group.

Still regarding the out-of-hospital environment in Brazil, the outcome is related to the rhythm of CPA. Shockable rhythms account for 80% of cases, with a survival rate of 50% to 70%, while non-shockable rhythms have a survival rate of less than 17% at all ages. A meta-analysis that included 141 studies conducted in North America, Europe, Asia, and Oceania on CPA in adults in the out-of-hospital setting found spontaneous circulation return rates of 29.7%, with a survival rate of less than 10%.^22 - 27^The literature still lacks similar data related to the pediatric age group.

The higher mortality in hospital environments, which are generally equipped with structural and human resources to perform CPR, draws attention. A study conducted in the USA with the pediatric age group showed a mortality rate of more than 60% due to CPA in hospitals.^22^In addition, as shown in a Brazilian observational study including CPR care delivered both on individual and team levels in a pediatric hospital, there is low adherence to the Pediatric Advanced Life Support protocol among health care professionals; this information is aligned with the mortality results found in the present study, since the quality of resuscitation directly impacts the survival of these individuals.^18^

The comparative analysis between primary causes of death and CPA as a notified event in a population sample is pioneering in the scientific literature and allows the understanding of comorbidities and etiologies most associated with this event, tracing a profile of individuals at greater risk of presenting CPA and progressing to death, so that these deaths can be prevented. Still, it is necessary to know the most common sequence of events that culminates in such deaths so that they can be prevented more specifically.

Limitations of this study include the use of secondary data and CDs filled with incomplete information. However, recent studies have shown an improvement in the quality of death coding in Brazil and a decrease in the use of garbage code, especially in the last 20 years.^23^These are the available data with the greatest impact on health in Brazil; additionally, they portray our population, justifying their widespread use in Brazilian scientific literature.

## Conclusion

The highest mortality rates from primary causes of death below the age of 20 years in Brazil from 1996 to 2019 were perinatal and external causes. When we evaluated multiple causes of death, the main primary causes related to CPA were respiratory, hematologic, and neoplastic diseases. Most deaths occurred in the hospital environment. A greater understanding of the chain of events in these deaths and the expansion and improvement of teaching strategies in pediatric CPA, aimed mainly at health care professionals, are necessary.

## Supplemental Materials

See the Supplemental Figure 1, please click here .

See the Supplemental Figure 2, please click here .

See the Supplemental Figure 3, please click here .

See the Supplemental Figure 4, please click here .

See the Supplemental Figure 5, please click here .

See the Supplemental Figure 6, please click here .
